# Complexity-Based Measures of Heart Rate Dynamics in Older Adults Following Long- and Short-Term Tai Chi Training: Cross-sectional and Randomized Trial Studies

**DOI:** 10.1038/s41598-019-43602-y

**Published:** 2019-05-16

**Authors:** Yan Ma, Chiu-wen Wu, Chung-Kang Peng, Andrew Ahn, Suzanne M. Bertisch, Lewis A. Lipsitz, Gloria Y. Yeh, Brad Manor, Vera Novak, Jeffrey M. Hausdorff, Brian Gow, Peter M. Wayne

**Affiliations:** 1 Division of Interdisciplinary Medicine and Biotechnology, Department of Medicine, Beth Israel Deaconess Medical Center, Harvard Medical School, Boston, MA United States; 2 National Taiwan Normal University, Department of Mechatronic Engineering, Taipei, Taiwan; 3 Division of General Medicine and Primary Care, Beth Israel Deaconess Medical Center, Harvard Medical School, Boston, MA United States; 4 Department of Pulmonary, Critical Care, and Sleep Medicine, Beth Israel Deaconess Medical Center, Harvard Medical School, Boston, MA United States; 5 Division of Gerontology, Beth Israel Deaconess Medical Center, Harvard Medical School, Boston, MA United States; 6 Hinda and Arthur Marcus Institute for Aging Research, Hebrew SeniorLife, Roslindale, MA United States; 7 Department of Neurology, Beth Israel Deaconess Medical Center, Harvard Medical School, Boston, MA United States; 8 Center for the Study of Movement, Cognition, and Mobility, Neurological Institute, Tel Aviv Sourasky Medical Center, Tel Aviv, Israel; 9 Department of Physical Therapy, Sackler Faculty of Medicine and Sagol School of Neuroscience, Tel Aviv University, Tel Aviv, Israel; 10 Rush Alzheimer’s Disease Center and Department of Orthopaedic Surgery, Rush University Medical Center, Chicago, Illinois United States; 11 Osher Center for Integrative Medicine, Brigham and Women’s Hospital, Harvard Medical School, Boston, MA United States

**Keywords:** Biomarkers, Cardiology, Health care

## Abstract

Measures characterizing the complexity of heart rate (HR) dynamics have been informative in predicting age- and disease-related decline in cardiovascular health, but few studies have evaluated whether mind-body exercise can impact HR complexity. This study evaluated the effects of long-term Tai Chi (TC) practice on the complexity of HR dynamics using an observational comparison of TC experts and age- and gender-matched TC-naïve individuals. Shorter-term effects of TC were assessed by randomly assigning TC-naïve participants to either TC group to receive six months of TC training or to a waitlist control group. 23 TC experts (age = 63.3 ± 8.0 y; 24.6 ± 12.0 y TC experience) and 52 TC-naïve (age = 64.3 ± 7.7 y) were enrolled. In cross-sectional analyses, TC experts had a higher overall complexity index (CI, p = 0.004) and higher entropy at multiple individual time scales (p < 0.05); these findings persisted in models accounting for age, gender, body mass index (BMI), and physical activity levels. Longitudinal changes in complexity index did not differ significantly following random assignment to six months of TC vs. a waitlist control; however, within the TC group, complexity at select time scales showed statistically non-significant trends toward increases. Our study supports that longer-term TC mind-body training may be associated with increased complexity of HR dynamics.

## Introduction

Human heart beat dynamics are influenced by the interaction of numerous factors, including the parasympathetic and sympathetic branches of the autonomic nervous system, hormones, body temperature, physical activity, digestion and circadian rhythms^1^. These processes impact heart rate dynamics across a broad range of time scales. Consequently, heart rate dynamics are exceedingly complex^2^. Indeed, a large body of research supports the concept that healthy heart rate rhythms exhibit features of complex systems reflecting dynamic, non-stationary, nonlinear properties^3^. Research also demonstrates that the complexity of heart rate dynamics decreases with age and disease^4,5^. For example, studies using multiscale entropy (MSE)––one widely used metric of complexity––observe age-related decline in heart rate complexity in healthy adults above the age of 40 years^6,7^. Compared to healthy adults, lower MSE of heart rate dynamics has also been associated with the presence of cardiac disease^8–11^, post-surgical complications^12,13^, mood disorders^14,15^, heart failure^16,17^, and overall cardiovascular related mortality in elderly adults^18^. In many of these and other studies, complexity-based metrics of heart rate dynamics have demonstrated better prognostic power compared with traditional measures of heart rate variability (HRV). However, to date, little research has evaluated whether the loss of cardiovascular physiological complexity associated with aging and age-related disease can be attenuated or restored with non-pharmacological interventions.

Tai Chi is a multimodal mind-body exercise with historical roots in traditional Chinese medicine. Its goal of impacting multiple physiological systems and integrating their dynamics make it especially well-suited for evaluation within a complexity framework^19–21^. Tai Chi is reported to improve or attenuate decline in many age-related health issues, including cardiovascular health^22,23^. Meta-analyses and systematic reviews support that Tai Chi may benefit multiple risk factors and clinically relevant outcomes related to cardiovascular health including: lowering systolic and diastolic blood pressure^24^; lowering levels of triglycerides, low-density lipoprotein (LDL), and serum B-type natriuretic peptide (BNP)^25,26^; and improving exercise capacity^27–30^, disease-related quality of life^31^, mood^32^, sleep quality^33^, and exercise self-efficacy^27,34^. Tai Chi may also reduce inflammatory markers known to contribute to coronary artery disease (CAD)^35,36^. A handful of smaller studies have also evaluated the impact of Tai Chi on a variety of time and frequency domain measures of HRV, however, the conclusions of these studies are mixed and difficult to interpret^37–41^. It remains unknown whether Tai Chi can impact complexity-based measures of heart rate dynamics.

As part of a larger study evaluating the impact of Tai Chi^19^ on physiological complexity and healthy aging, the current work evaluates the impact of both long- and short-term Tai Chi training on MSE of heart rate dynamics monitored during 5 minute bouts of natural breathing under controlled experimental conditions. Potential long-term effects of TC training were evaluated through cross-sectional comparisons of healthy Tai Chi naïve adults and age- and gender-matched expert Tai Chi practitioners. Tai Chi short-term effects were evaluated by random assignment of the Tai Chi naïve healthy participants to either six months of Tai Chi plus usual care or to usual care alone. Based on our previously completed studies, we hypothesized that (1) Tai Chi experts may have greater MSE of heart rate dynamics, compared to age- and gender-matched controls; and (2) Tai Chi naïve older adults who were randomized to six months of Tai Chi would subsequently show greater MSE of heart rate dynamics compared to a wait-list control. For comparison with MSE, traditional time domain measures of HRV were also evaluated.

## Results

### Participant characteristics and flow through the trial

Recruitment of both Tai Chi experts (N = 27) and Tai Chi naïve individuals (N = 60) took place between March 2011 and March 2013, and follow-up assessments were completed in September 2013. Of the 27 Tai Chi experts, data from 4 were discarded due to artifacts in the ECG signals providing an analyzed sample of 23 individuals. The average training experience of Tai Chi experts was 24.6 ± 12.0 years (median 20 yrs, range 10–50 yrs). Tai Chi naïve subjects were overall well-matched with Tai Chi experts in respect to age, reported presence of hypertension, and global cognitive status. However, Tai Chi experts showed higher physical activity levels, lower body mass index (BMI), and were represented by a slightly higher proportion of men and Asians. Tai Chi naïve subjects who were subsequently randomized to the Tai Chi group versus the usual care control group were comparable at baseline (Table 1).

**Table 1:** Baseline characteristics.

	Randomized groups		Observational group
	Usual care (n = 26)	Tai Chi (n = 26)	Tai Chi experts (n = 23)
Age, years	64.85 ± 7.62	64.15 ± 7.69	63.30 ± 8.03
Gender, n (%)			
Female	16 (61.5%)	18 (69.2%)	12 (52.2%)
Male	10 (38.5%)	8 (30.8%)	11 (47.8%)
BMI, kg/m^2^	27.89 ± 7.83	27.14 ± 5.46	23.79 ± 3.20^a^
Race, n (%)			
White	23 (88.5%)	24 (92.3%)	18 (78.3%)
African-American	3 (11.5%)	0 (0%)	1 (4.3%)
Asian	0 (%)	2 (7.7%)	4 (17.4%)
Ethnicity, n (%)			
Non-Hispanic/Non-Latino	26 (100%)	25 (96.2%)	22 (95.7%)
Hispanic/Latino	0 (0%)	1 (3.8%)	1 (4.3%)
Education, years	16.17 ± 3.14	17.57 ± 3.33	17.75 ± 2.07
Mini Mental State Exam scores	29.15 ± 0.83	28.96 ± 1.25	29.00 ± 1.08
Physical activity level^b^	4.00 ± 2.26	4.54 ± 2.2	5.74 ± 2.03^a^
Hypertension, n (%)	16 (61.5%)	12 (46.2%)	13 (56.5%)
SBP (mmHg)	126.19 ± 14.37	126.58 ± 15.70	125.68 ± 16.84
DBP (mmHg)	73.23 ± 8.33	72.88 ± 7.05	75.27 ± 10.28
Heart beat intervals (s)	0.98 ± 0.13	1.01 ± 0.12	0.99 ± 0.14

^a^Cross-sectional comparisons between Tai Chi experts and Tai Chi naïve adults at baseline differed significantly in BMI (p = 0.013) and physical activity level (p = 0.021).

^b^4 = Run about 1 mile/week OR walk about 1.3 miles/week OR spend about 30 min/week in comparable physical activity; 5 = Run about 1 to 5 miles per week OR walk 1.3 to 6 miles per week OR spend 30 to 60 minutes per week in comparable physical activity; 6 = run about 6–10 miles/week OR walk 7–13 miles/week OR spend 1–3 h/week in comparable physical activity. Abbreviations: BMI, body mass index; SBP, systolic blood pressure; DBP, diastolic blood pressure.

**Figure 1:**
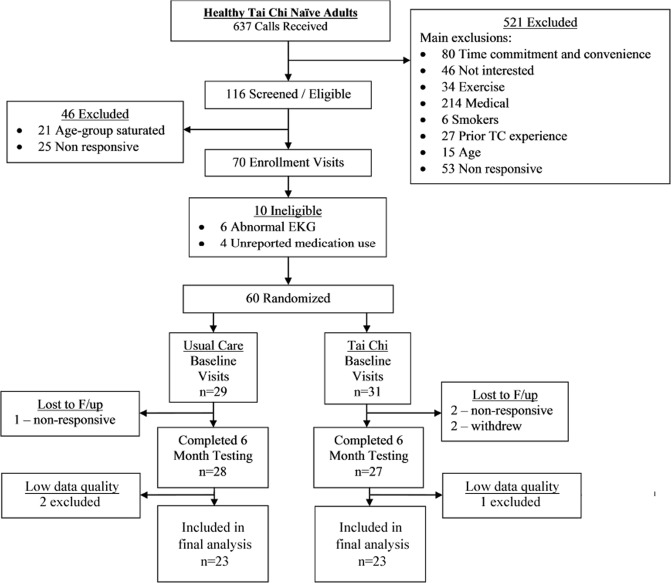
Participant flow through the randomized trial sub-study.

Figure 1 summarizes the flow of participants in the randomized controlled trial included in this study. As a previously published study reported^42^, sixty healthy adults were enrolled. Among them, 28 of 29 (97%) individuals in the Tai Chi group, and 27 of 31 (87%) individuals in the usual care group completed the 6-month follow-up assessment. Data from 8 Tai Chi naïve participants were excluded due to the loss of follow-up (n = 5) or the low ECG quality (artifacts or excessive ectopic beats, n = 3), resulting in 26 subjects randomized to each of the Tai Chi and waitlist control groups with analyzable baseline and 6 month data.

### Cross-sectional comparisons

**Figure 2:**
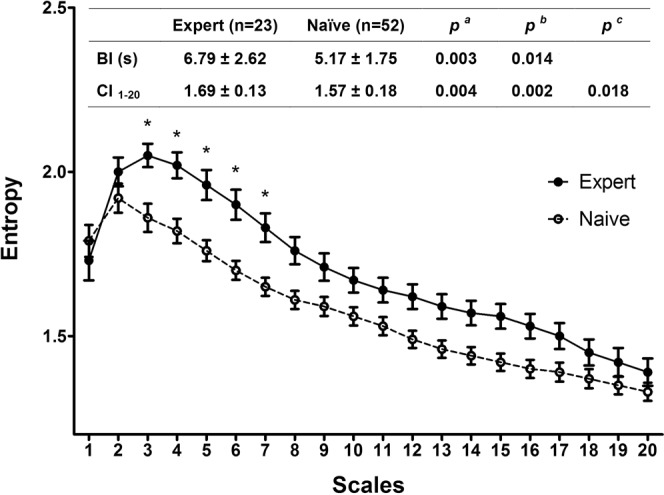
Baseline complexity compared between experts and naïve, presented by mean and standard error of the group. (**a**) Unadjusted *p* values calculated from t-test. (**b**) Adjusted *p* values calculated from linear model adjusting for age, BMI, and physical activity. (**c**) Adjusted *p* values calculated from linear model adjusting for age, BMI, physical activity and BI. Abbreviations: BI, intervals of breathing peaks; NN, normal-to-normal heartbeat intervals; CI_1–20_, complexity index calculated by the mean entropy of 20 scales. Complexity index (CI) curves for Tai Chi experts and Tai Chi naïve evaluated cross-sectionally at baseline. Values represent mean and standard error for each scale. Embedded table summarizes statistics for comparisons. Significantly different comparisons at individual scales (following Bonferroni adjustments) are denoted with an asterisk (*).

Compared to the novices, Tai Chi experts exhibited an overall higher MSE_mod_ complexity index over 20 scales (CI_1–20_) compared to Tai Chi naïve controls (CI_1–20_ of 1.69 ± 0.13 vs. 1.57 ± 0.18, p = 0.004) (Fig. 2). This group difference remained statistically significant in linear models adjusting for age, BMI, and physical activity (P = 0.002). Secondary analyses evaluating individual scales of the MSE_mod_ suggest significant differences at most scales (scale 3–17) in analyses not including Bonferroni adjustments for multiple comparisons. Differences at scales 3–7 remained significant following Bonferroni adjustments. Similar findings were observed in models adjusting for age, BMI, and physical activity. Tai Chi experts also exhibited longer breathing intervals (p = 0.003). After adjustment for age, BMI, activity level, and breathing intervals, between groups differences in complexity index were still significant (p = 0. 018).

In contrast, conventional time domain parameters did not show any difference between Tai Chi experts and naïve controls, even after adjusting for age, BMI, level of activity and breathing (Table 2).

**Table 2:** Cross-sectional comparison in HRV time domain parameters for Tai Chi experts vs. naïve individuals.

Outcome measures	Tai Chi expert (n = 23)	Tai Chi naïve (n = 52)	Unadjusted *p*-value^a^	Adjusted *p*-value^b^	Adjusted *p*-value^c^
HR (beats/min)	61.94 ± 10.04	61.17 ± 7.57	0.715	0.386	0.122
AVNN (ms)	991.48 ± 137.15	998.24 ± 124.09	0.835	0.419	0.178
SDNN (ms)	42.14 ± 12.93	45.03 ± 19.58	0.519	0383	0.142
RMSSD (ms)	23.91 ± 7.94	32.93 ± 23.21	0.074	0.061	0.163
pNN20 (%)	30.79 ± 14.52	36.80 ± 20.46	0.208	0.128	0.163
pNN50 (%)	5.05 ± 5.63	10.51 ± 16.07	0.118	0.075	0.209

^a^*p* values calculated from unadjusted linear regression model.

^b^*p* values calculated from linear model adjusting for age, BMI, and physical activity.

^c^*p* values calculated from linear model adjusting for age, BMI, physical activity, and breathing intervals.

Abbreviations: HR, heart rate; NN, normal-to-normal heartbeat intervals; AVNN, average NN intervals; SDNN, standard deviations of NN intervals; RMSSD, root mean square of the successive differences; pNN20 and pNN50, percentage of adjacent NN intervals that differ by greater than 20 and 50 milliseconds respectively.

### Longitudinal comparisons

**Figure 3:**
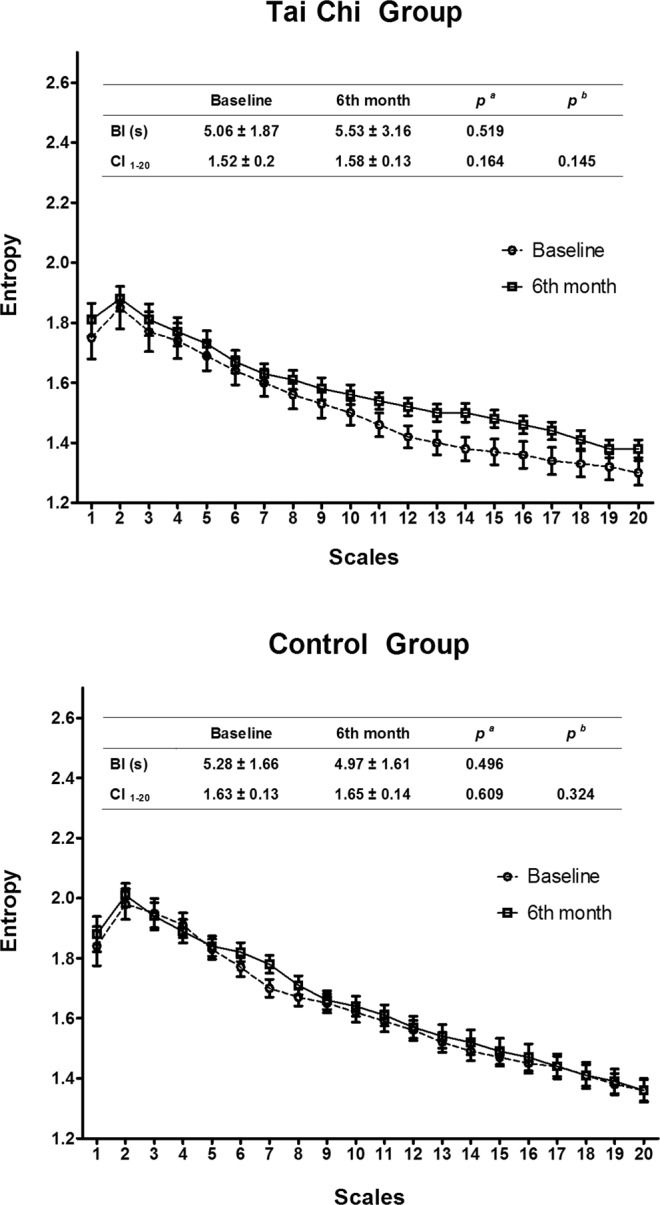
Pre- and post- comparisons at all scales of Tai Chi and control group. (**a**) Unadjusted p values calculated from t-test. (**b**) Adjusted *p* values calculated from linear model adjusting for BI. Abbreviations: BI, intervals of breathing peaks.

There was a trend towards a greater increase in the overall MSE_mod_ complexity index (CI_1–20_) in healthy TC naïve adults randomly assigned to 6 months of Tai Chi (1.52 ± 0.2 to 1.58 ± 0.13) versus usual care group (1.63 ± 0.13 to 1.65 ± 0.14). However, within (baseline vs. 6 month) and between (Tai Chi vs. naïve) group differences were not statistically significant based on all linear models evaluated. When MSE_mod_ was further explored and analyzed on individual scales (Fig. 3), within group differences in those randomized to Tai Chi exhibited trends towards increased entropy on a subset of larger scales (scales 11–17), with differences being statistically significant at scales 13 (p = 0.047), 14 (p = 0.024) and 15 (p = 0.034) in analyses not including Bonferroni adjustments for multiple comparisons. However, after adjustments for multiple comparisons, these differences were no longer significant. In contrast, no notable changes in individual complexity scales in the control group were observed after 6 months.

There was a trend towards longitudinal increases in breathing intervals (i.e. slower breathing) in the Tai Chi group (5.0 ± 1.87 to 5.53 ± 3.16 seconds), compared to the control group (5.28 ± 1.66 to 4.97 ± 1.61 seconds). However, within- and between-group differences were not statistically significant in any of the models.

In contrast to MSE_mod_, conventional time domain parameters of HRV did not show any trends when longitudinal comparisons were made between those randomly assigned to Tai Chi versus usual care (Table 3).

**Table 3:** Longitudinal comparisons in HRV time domain parameters for older adults randomly assigned to 6 months of Tai Chi vs. usual care.

Outcome measures	Tai Chi (n = 26)			Usual care (n = 26)			Between groups			
	Baseline	6th month	*p* ^a^	Baseline	6th month	*p* ^a^	*p*^b^ for baseline	*p*^b^ for 6^th^ month	Group *p*^c^	Group * Time *p*^c^
HR (beats/min)	60.21 ± 7.33	62.42 ± 12.75	0.455	62.08 ± 7.83	64.27 ± 12.36	0.449	0.384	0.597	0.975	0.497
AVNN (ms)	1013.64 ± 123.67	991.22 ± 149.22	0.563	983.42 ± 125.08	959.07 ± 136.13	0.505	0.390	0.421	0.703	0.574
SDNN (ms)	49.72 ± 21.03	45.10 ± 20.71	0.433	40.53 ± 17.29	36.53 ± 13.68	0.359	0.094	0.084	0.206	0.464
RMSSD (ms)	39.57 ± 29.48	33.49 ± 24.50	0.426	26.55 ± 12.50	23.52 ± 10.10	0.341	0.044*	0.061	0.038*	0.409
pNN20 (%)	41.46 ± 24.12	39.58 ± 24.38	0.783	32.32 ± 15.36	29.47 ± 16.14	0.516	0.112	0.084	0.206	0.822
pNN50 (%)	15.66 ± 20.44	12.03 ± 16.94	0.492	5.56 ± 7.97	4.34 ± 5.67	0.527	0.023*	0.033*	0.026*	0.543

^a^*p* values calculated from unadjusted linear regression model.

^b^*p* values calculated from unadjusted linear regression model.

^c^*p* values calculated from linear model adjusting for age and gender.

Abbreviations: HR, heart rate; NN, normal-to-normal heartbeat intervals; AVNN, average NN intervals; SDNN, standard deviations of NN intervals; RMSSD, root mean square of the successive differences; pNN20 and pNN50, percentage of adjacent NN intervals that differ by greater than 20 and 50 milliseconds respectively.

## Discussion

In our cross-sectional comparisons, we observed that long-term training in TC is associated with higher (i.e., more complex) MSE_mod_ of heart rate dynamics across a wide range of scales. Importantly, statistical differences between TC experts and TC naive controls persisted even when multiple potential confounders were taken into account. In our randomized trial, we observed that a 6-month exposure to Tai Chi resulted in statistically non-significant within-group trends towards increases in MSE at a subset of MSE scales, however, between-group differences in longitudinal changes were not significant. Of note, no differences were found in average heart rate or conventional time domain HRV parameters for either long-term or short-term Tai Chi exposures. Together, these findings add to a growing body of research supporting that Tai Chi can have positive effects on markers and risk factors associated with cardiovascular health^43,44^. Moreover, along with other recent studies reporting an association of Tai Chi training with enhanced physiological complexity of balance^45,46^ and gait dynamics^42^, our findings support the general value of complexity-based indices for informing the impact of Tai Chi on health.

Ageing and disease are commonly associated with multi-system impairment^4^, resulting in complex changes in physiological dynamics and function^5,47^. For this reason, it has been argued that complexity-based metrics that provide indices related to the magnitude of physiological ‘cross-talk’, feedback and multi-scale regulation, might serve as sensitive and meaningful biomarkers of age- and disease-related decline^5^. While many studies have confirmed the value of complexity-based measures for characterizing and predicting age- and disease-related decline based on the dynamics of a variety of physiological signals such as heart rate^48–50^, blood pressure^51–53^, center of pressure^45,46,54^, stride time^42^, and gait variability^55^, surprisingly few studies to date have evaluated the potential of interventions to attenuate or restore age- or disease-related decline in complexity. With respect to heart rate dynamics, one 8-week study evaluated administration of the anti-psychotic clozapine for treatment-resistant schizophrenia. This study reported that positive clinical responses were correlated with increases in complexity of heart rate dynamics measured with approximate and sample entropy^56^. Another small study in children affected by a spectrum of neurological disorders reported that short-term exposure to hippotherapy (therapeutic horse riding) led to greater post-treatment sample entropy of HRV^57^. A third non-controlled study evaluating a meditation technique reported higher sample entropy in heart beat dynamics following an individual meditation session^58^. Our study extends this small body of research by both evaluating a novel mind-body exercise intervention, and by demonstrating potential impacts on heart rate dynamics well beyond acute, single-session exposure effects.

Compared to robust cross-sectional differences observed between Tai Chi experts and naive controls in MSE_mod_, we observed only modest and statistically non-significant differences over time in MSE_mod_ for TC naive adult participants randomized to six months of TC training vs. a waitlist control. This smaller effect may reflect inadequate dose (too short a duration and/or too low an intensity of training) of Tai Chi to alter fundamental HR dynamics. The fact that the population in our study was very healthy and already physically active may have further contributed to the observed small effect. These findings parallel those observed in gait and balance dynamics outcomes in the same population, with only modest effects observed after 6 months of Tai Chi exposure^42,45,55,59^. Future studies assessing the impact of TC practice on HR complexity might consider evaluating less healthy populations, and administering greater intensities and longer exposures of Tai Chi training.

The complexity of physiologic control systems is believed to be important, as it affords the organism resilience leading to better adaptation to a specific task or an external stress^4,60^. To date, existing evidence to support this theory is very limited. In elderly people, studies have shown that the magnitude of complexity exhibited within center of pressure dynamics during quiet standing is correlated with the ability to balance while challenged with a mental dual task (i.e., counting backwards)^61^. With regards to heart rate dynamic measures evaluated in this study, future studies might also explore if the Tai Chi-related increases in MSE_mod_ are associated with enhanced resiliency in heart rate or blood pressure responses during sit-to-stand challenges or acute bouts of aerobic exercise.

In contrast to the observed associations between Tai Chi training and complexity of heart rate dynamics, we observed no long- or short-term effects of Tai Chi on traditional time domain measures of HRV. Indices of HRV based on time domain methods have been shown to be predictive of adverse outcomes in patients with heart disease^62–64^ and in the general population. However, the evidence that Tai Chi and related mind-body studies enhance time domain measures of HRV are quite mixed^37^, and many rely only on short-term (post single session) acute effects^39,41,65,66^. In studies evaluating the longer-term impact of Tai Chi training, some studies report increased HRV^67,68^, while others report no effect^27,40,69^, or even reduced variability^70^. Part of this heterogeneity may be attributed to the confounding effects of respiratory rate on traditional HRV measure. It is well established that changes in respiratory frequency can affect tidal volume (e.g., lower respiratory rate is associated with greater tidal volumes) and thus the amplitude of heart rate oscillations via a respiratory sinus arrhythmia (RSA) mechanism. Traditionally, this vagally-mediated process is captured in the high frequency (HF) component of HRV. However, in our cohort of Tai Chi experts, the mean breathing interval was 6.79 s corresponding to a rate of 0.147 Hz or 8.8 breaths/min, compared to the 5.17 seconds or 0.193 Hz (11.6 breaths/min) seen in the control group. As a consequence, the vagally-mediated RSA would be categorized into the low frequency (LF) component (0.04–0.15 Hz) of HRV for many within the Tai Chi group, whereas it would be appropriately categorized into the HF HRV component (0.15–0.40 Hz) for most in the control group. The reduced respiratory frequency observed in the Tai Chi group not only increases RSA-associated HRV but can also lead to mis-categorization of the parasympathetic nervous activity into the LF band. This confounding effect of respiration on HRV may explain the variability in the reported effects of Tai Chi on HRV, and indeed, the majority of studies evaluating the impact of Tai Chi on HRV do not account for breathing^65–67,69^.

Multiscale Entropy, on the other hand, remains relatively robust to respiratory rate as evidenced by the sustained statistically significant difference in MSE_mod_, even after accounting for differences in breathing intervals in the Tai Chi experts group and TC naïve group (Fig. 2). The MSE_mod_ complexity index (CI_1–20_) employed in this study is essentially a composite score that theoretically represents the summative effects of physiological control (including, but not exclusive to, respiratory contributions) of HRV in the time domain of ~0.8 to 25 seconds (depending on the mean HR). As a result, the application of MSE does not require an additional process to correct for respiratory confounding – such as paced breathing which mandates an artificially forced condition onto the body. In addition, the scales utilized in MSE are not a function of time, but rather a function of the number of RR (or NN) intervals. For instance, Scale 2 SampEn is derived by utilizing the coarse-grained time series for averages of *two* consecutive RR-intervals. MSE utilizes heart beat (and not time) as the frame of reference, and thus avoids the need for an arbitrarily-chosen range of frequencies to capture specific physiological processes. Whether this approach yields more physiologically-consonant results compared to a time-based approach remains to be further tested. Finally, MSE has the additional benefit of extracting information from the sequential order of RR-intervals. Traditional HRV measures, both time- and frequency-based, are linear measures that simply reveal the distribution of the data, whether it be the spectral power or number of RR-intervals in a specific time range. The failure to account for the sequential order (i.e., what RR interval follows a previous one) may explain why traditional HRV measures are not able to effectively capture differences in health-states when compared to complexity-based metrics such as MSE.

This study may have a number of limitations. First, sample sizes were small for both cross-sectional comparisons and randomized controlled trial (RCT). In addition, as with any observational study, comparisons may be confounded by differences between groups other than exposure to Tai Chi. Although our statistical models accounted for multiple confounders, there may still be other factors not measured or included. There was not an active control in our RCT, thus is possible that observed effects were due to social support and/or placebogenic beliefs. Future studies evaluating Tai Chi on complexity measures of heart rate dynamics should consider employing active comparative controls. Additionally, it may be that six months of Tai Chi training is an insufficient dose to significantly change heart rate dynamics in healthy adults. Finally, given the 5-minute duration of the RR-interval data, the physiological significance of MSE_mod_ at higher scales (up to Scale 20) remain unclear, and the full dynamics of the heart rate variability at these lower frequencies may be under-represented in our results and analyses.

## Conclusions

Long-term Tai Chi mind-body training may be associated with increased complexity of HR dynamics in healthy adults, as measured with MSE_mod_. Compared to traditional time domain HRV measures of cardiovascular health, MSE_mod_ appears to be a more sensitive in evaluating the impact of a multi-modal mind-body exercise.

## Materials and Methods

### Study Design

Design features of this study have been reported elsewhere^19,42,45,59^. This study was approved by the Institutional Review Boards of Beth Israel Deaconess Medical Center and Brigham and Women’s Hospital, and conducted in accordance with the relevant guidelines and regulations. The randomized trial component of this study was registered at ClinicalTrials.gov (NCT01340365, date of registration: April 22nd, 2011).

Our study design that integrated a two-arm RCT with an cross-sectional comparison group at baseline^19^. Reports of other outcomes from this study have already been published^34,42,45,55,59^: The Tai Chi naïve group was composed of 60 adults, age 50–79 years, and reporting no consistent training in Tai Chi in the prior 5 years. Exclusion criteria included: 1) chronic medical condition (e.g. cardiovascular disease, cancer; neurological conditions); 2) acute conditions requiring hospitalization in the prior 6 months; 3) inability to walk unassisted and continuously for 15 minutes; and 4) engagement in physical exercise four or more times in a given week. Eligible participants provided written informed consent and were randomized (1:1) to either 6 months of Tai Chi training in combination with usual healthcare, or usual healthcare alone. Randomization was age-stratified (50–59, 60–69, 70–79 years) utilizing a permuted-blocks scheme with randomly varying block sizes. Outcomes were assessed at baseline and 6 months. Staff overseeing and conducting the assessments were blinded to treatment assignment. Particpants assigned to the Tai Chi group were required to attend at least 2 study-sanctioned community-based Tai Chi classes each week and engage in self-practice at home for 30 min on 2 additional days each week^17^. Tai Chi class attendance monitored by instructors and compliance with home practice was tracked with self-completed logs.

The TC expert group included 27 adults (age 50–79 yrs) currently engaged in ongoing Tai Chi training, each with at least 5 years of experience. Other than the restriction on Tai Chi practice, procedures for identifying and enrolling Tai Chi experts were identical to the Tai Chi naïve group.

### Measurements

All testing was performed in the Syncope and Falls in the Elderly (SAFE) laboratory, located in Beth Israel Deaconess Medical Center (Boston, MA). Outcomes related to heart rate dynamics were just one components of a more comprehensive battery of tests (including balance^45^, gait^55^, and cognition^59^). Characterization of heart rate dynamics employing MSE was defined a priori as a primary outcome measure^19^. Tai Chi experts evaluated in longer-term comparisons were assessed at only one point in time. Tai Chi naive participants were assessed at baseline and at 6 months following randomization to either Tai Chi or a wait list control. For all subjects, demographic, anthropometric, and relevant medical histories data were obtained at screening visits.

Heart rate dynamics were assessed using a standardized testing protocol employed in prior studies^71,72^. Subjects were asked to refrain from caffeine on the day of testing. Following a 10-minute resting period, electrocardiogram (ECG) and respiratory signals from CO2 recordings were collected while participants laid supine and were instructed to breathe naturally for 5 minutes. ECG and respiratory signals were sampled at 500 Hz. Consecutive R peaks were detected from raw ECG recordings and the time series were extracted as RR intervals for analyses proposed in this study^50^. Ectopic beats were detected and removed from calculations as in previous studies^50^. ECG data with arrhythmias or artifacts that comprised greater than 5% of the total epoch were removed. Qualified clean data were used for analyses in the presented study. Respiration cycles were detected by identifying consecutive peaks of the respiratory signal, from which mean breathing intervals (BI) were calculated^73,74^. Breathing intervals were recorded to inform potential differences in heart rate complexity between participants with and without exposure to Tai Chi, which typically includes instruction in breathing. Since lower breathing frequencies are associated with increased tidal volumes, increased breathing intervals are typically associated with increased respiratory sinus arrhythmia, which may ultimately affect the overall HR complexity value.

### Estimate of Multiscale Entropy of Heart Rate Dynamics

Multiscale entropy (MSE) of HR dynamics utilizes Sample Entropy (SampEn) to quantify the degree of irregularity within a time-series (e.g. RR or NN intervals) across multiple time scales^45^. For relatively longer time series (e.g., hours), MSE first divides the original signal into non-overlapping segments of length *τ*, then averages the data points inside each segment, which is the “coarse-graining” process^48^. SampEn is calculated for each coarse-grained time series at different scales. The commonly used parameters for MSE are m = 2, r = 15% of RR intervals’ standard deviation. The traditional coarse-graining process shortens the time series data with each increasing time scale and thus limits the extent to which SampEn can accurately be calculated at larger time scales. Based on the data we collected in this study, we calculated MSE using a validated modified approach, referred to as modified multiscale entropy (MSE_mod_)^75,76^. MSE_mod_ was developed to specifically be suitable for shorter-term time series and to provide stable, reliable, and precise estimates for short-term time series. MSE_mod_ adheres to most of the conventional MSE algorithm, but replaces the coarse-graining procedure with a moving average procedure, and a time delay was incorporated for constructing template vectors in calculating sample entropy^75^. This ensures that sufficient data are maintained for entropy calculation, even at large scales (i.e., lower frequencies) and for shorter time series data (Supplementary file 1). In this study, parameters were set as m = 2, r = 15% of RR intervals’ standard deviation. Entropies were calculated up to 20 scales, and a complexity index was calculated as mean of the entropy values of scale 1–20 (CI_1–20_).

### Time Domain Parameters of Heart Rate Variability

Analyzed time domain parameters included mean NN intervals (NN), standard deviation of NN intervals (SDNN), mean heart rate (HR), root mean square of the successive differences (RMSSD), percentage of adjacent NN intervals that differ by greater than 20 milliseconds (pNN20) and 50 milliseconds (pNN50).

### Baseline characteristics

Sociodemographic variables collected at baseline included age, height, weight, race and ethnicity, medical history, current medication use, diet, smoking and alcohol history, education, marital status, employment status, and level of physical activity. Physical activity level at baseline was evaluated by the physical activity status scale (PASS)^77^. Baseline global cognitive function was evaluated using the Mini Mental State Exam^59^.

### Statistical analyses

Statistical analyses were performed using SPSS 19.0 (IBM SPSS Statistics). Continuous data was tested for normality of the distribution, and descriptive statistics were presented as mean ± standard deviation for normal distributed data. Categorical data were reported as umber (percentage). Between-group comparisons of baseline characteristics were assessed by chi-square test for categorical variables. Cross-sectional comparisons were assessed by linear regression models including three levels of potential confounders between TC experts and TC naïve control subjects. Model 1 was based on raw unadjusted data. Model 2 incorporated the potential confounding effects of age, BMI, and levels of physical activity following analytic methods employed to evaluate other outcomes based on this same experimental design and study population^42,45,55,59^. Model 3 included breathing intervals in addition to Model 2 variables.

Longitudinal comparisons in the randomized trial of this study were evaluated by baseline-adjusted linear regression models including three levels of potential confounders. Model 1 was based on raw unadjusted data. Model 2 included the potential confounders of age and gender as in our prior studies^42,45,55,59^. Model 3 included breathing intervals in addition to Model 2 variables.

## Supplementary information

Supplementary File
