# Supplementary material for: Complexity-Based Measures of Heart Rate Dynamics in Older Adults Following Long- and Short-Term Tai Chi Training: Cross-sectional and Randomized Trial Studies
Source: Sci Rep. 2019 May 16;9:7500. doi: 10.1038/s41598-019-43602-y (PMC6522618; doi:10.1038/s41598-019-43602-y)
Supplement: Supplementary file 1 — Supplementary File [file 41598_2019_43602_MOESM1_ESM.pdf]

Complexity-Based Measures of Heart Rate Dynamics in Older Adults Following Long- and Short-Term Tai Chi Training: Cross-sectional and Randomized Trial Studies

Yan Ma[1], Chiu-wen Wu[2], Chung-Kang Peng[1], Andrew Ahn[3], Suzanne M. Bertisch[4], Lewis A. Lipsitz[5,6], Gloria Y. Yeh[3, 9], Brad Manor[5, 6], Vera Novak[7], Jeffrey M. Hausdorff[8], Brian Gow[1,9], Peter M. Wayne[9]

1. Division of Interdisciplinary Medicine and Biotechnology, Department of Medicine, Beth Israel Deaconess Medical Center, Harvard Medical School, Boston, MA, United States
2. National Taiwan Normal University, Department of Mechatronic Engineering, Taipei, Taiwan
3. Division of General Medicine and Primary Care, Beth Israel Deaconess Medical Center, Harvard Medical School, Boston, MA, United States
4. Department of Pulmonary, Critical Care, and Sleep Medicine, Beth Israel Deaconess Medical Center, Harvard Medical School, Boston, MA, United States
5. Division of Gerontology, Beth Israel Deaconess Medical Center, Harvard Medical School, Boston, MA, United States
6. Hinda and Arthur Marcus Institute for Aging Research, Hebrew SeniorLife, Roslindale, MA, United States
7. Department of Neurology, Beth Israel Deaconess Medical Center, Harvard Medical School, Boston, MA, United States
8. Center for the Study of Movement, Cognition, and Mobility, Neurological Institute, Tel Aviv Sourasky Medical Center; Department of Physical Therapy, Sackler Faculty of Medicine and Sagol School of Neuroscience, Tel Aviv University, Israel; Rush Alzheimer's Disease Center and Department of Orthopaedic Surgery, Rush University Medical Center, Chicago, Illinois, United States

9. Osher Center for Integrative Medicine, Brigham and Women's Hospital, Harvard Medical School, Boston, MA, United States

Supplemental file: Validation of MSE_{mod} method

To validate whether the MSE_{mod} method is applicable to short time series like the data used in our study, we used pink noise (Gaussian distributed 1/f noise to simulate healthy subjects) and white noise (to simulate AF patients) to run simulations:

- Data: Gaussian distributed 1/f noise vs Gaussian distributed white noise;
- Data length: N = 2000, 1000, 500, 300, 200, and 100;
- MSE_{mod} parameter settings: m=2, r=0.15;
- Approach: We ran each data set 1000 times and calculated the mean entropy at each scale, the standard deviation of the mean, and the probability of measurement failure.

MSE calculates sample entropy on different scales: In the process of SampEn calculations, errors may occur when there are not enough data points, leading to A=0 or A=B=0. When A=0, SampEn=Inf; When A=B=0, SampEn=NaN; these are not meaningful results. We calculate the probability of measurement failure as the probability of such events.

We also used MSE_{mod} to compare two signals and test whether the algorithm is stable enough to distinguish between pink and white noise as the data length is decreased. We have listed all the results here:

Table 1(c) and 2(c) indicate that MSE_{mod} is applicable to short data since the probability of measurement failure is very low for pink noise. Table 3 indicates that MSE_{mod} is sensitive enough to tell the difference between pink noise and white even when the data length is N=100.

Results:

Data: Gaussian distributed 1/f noise vs Gaussian distributed white noise

Data length: N = 2000, 1000, 500, 300, 200, and 100

MSE_{mod} parameters setting: m=2, r =0.15

Run 1000 times for mean, standard deviation and probability of measure failure.

Table 1: Gaussian distributed 1/f noise

Table 1(a) Mean

Scale	1	2	3	4	5	6	7	8	9	10	11	12	13	14	15	16	17	18	19	20
N=2000	1.98	1.94	1.92	1.90	1.90	1.90	1.89	1.88	1.89	1.89	1.89	1.89	1.88	1.89	1.89	1.88	1.88	1.89	1.89	1.89
N=1000	2.02	1.98	1.96	1.95	1.94	1.94	1.94	1.93	1.94	1.94	1.94	1.95	1.94	1.94	1.94	1.94	1.95	1.95	1.96	1.96
N=500	2.07	2.03	2.01	2.00	2.00	1.99	2.00	1.98	2.00	2.02	2.03	2.03	2.03	2.04	2.05	2.05	2.07	2.09	2.10	2.11
N=300	2.11	2.09	2.06	2.05	2.05	2.05	2.07	2.07	2.09	2.13	2.16	2.19	2.22	2.27	2.29	2.30	2.36	2.37	2.40	2.42
N=200	2.16	2.14	2.12	2.11	2.12	2.13	2.18	2.19	2.25	2.32	2.38	2.38	2.40	2.39	2.43	2.44	2.42	2.37	2.38	2.35
N=100	2.32	2.29	2.29	2.29	2.31	2.29	2.27	2.21	2.20	2.11	2.09	1.96	1.85	1.75	1.69	1.58	1.50	1.57	1.38	1.32

Table 1(b) Standard deviation

Scale	1	2	3	4	5	6	7	8	9	10	11	12	13	14	15	16	17	18	19	20
N=2000	0.09	0.09	0.09	0.09	0.09	0.09	0.09	0.10	0.10	0.10	0.10	0.10	0.10	0.10	0.10	0.10	0.11	0.11	0.11	0.11
N=1000	0.10	0.10	0.10	0.11	0.11	0.11	0.12	0.13	0.14	0.14	0.14	0.15	0.15	0.15	0.16	0.17	0.18	0.19	0.20	0.21
N=500	0.13	0.13	0.14	0.14	0.15	0.16	0.18	0.19	0.21	0.22	0.25	0.26	0.27	0.29	0.31	0.32	0.35	0.36	0.38	0.41
N=300	0.18	0.17	0.18	0.19	0.20	0.23	0.26	0.30	0.32	0.36	0.42	0.50	0.55	0.62	0.67	0.68	0.78	0.76	0.84	0.91
N=200	0.25	0.24	0.25	0.27	0.30	0.36	0.42	0.47	0.55	0.63	0.71	0.77	0.81	0.81	0.88	0.91	0.94	0.91	0.98	1.05
N=100	0.50	0.48	0.53	0.56	0.60	0.62	0.66	0.68	0.73	0.77	0.82	0.82	0.85	0.87	0.97	0.99	1.04	1.07	0.99	1.01

Table 1(c) Probability of measure failure

Scale	1	2	3	4	5	6	7	8	9	10	11	12	13	14	15	16	17	18	19	20
N=2000	0.0%	0.0%	0.0%	0.0%	0.0%	0.0%	0.0%	0.0%	0.0%	0.0%	0.0%	0.0%	0.0%	0.0%	0.0%	0.0%	0.0%	0.0%	0.0%	0.0%
N=1000	0.0%	0.0%	0.0%	0.0%	0.0%	0.0%	0.0%	0.0%	0.0%	0.0%	0.0%	0.0%	0.0%	0.0%	0.0%	0.0%	0.0%	0.0%	0.0%	0.0%
N=500	0.0%	0.0%	0.0%	0.0%	0.0%	0.0%	0.0%	0.0%	0.0%	0.0%	0.0%	0.0%	0.0%	0.0%	0.0%	0.0%	0.0%	0.0%	0.0%	0.0%
N=300	0.0%	0.0%	0.0%	0.0%	0.0%	0.0%	0.0%	0.0%	0.0%	0.0%	0.0%	0.0%	0.0%	0.0%	0.9%	1.9%	2.5%	4.2%	5.3%	6.2%
N=200	0.0%	0.0%	0.0%	0.0%	0.0%	0.0%	0.0%	0.2%	0.1%	0.6%	1.9%	5.7%	7.8%	13.2%	16.7%	20.6%	26.6%	31.0%	34.9%	41.2%
N=100	1.0%	1.8%	2.4%	3.2%	8.8%	13.2%	21.7%	31.1%	41.4%	53.1%	59.2%	64.5%	71.9%	77.2%	82.3%	85.0%	87.7%	90.0%	91.4%	93.4%

Table 2: Gaussian distributed white noise

Table 2(a) Mean

Scale	1	2	3	4	5	6	7	8	9	10	11	12	13	14	15	16	17	18	19	20
N=2000	2.48	2.13	1.93	1.79	1.68	1.59	1.52	1.45	1.40	1.34	1.30	1.26	1.23	1.19	1.16	1.13	1.10	1.08	1.05	1.03
N=1000	2.48	2.13	1.93	1.79	1.68	1.59	1.52	1.46	1.40	1.35	1.31	1.27	1.23	1.20	1.17	1.14	1.11	1.09	1.06	1.04
N=500	2.49	2.13	1.93	1.80	1.68	1.60	1.53	1.46	1.41	1.36	1.32	1.28	1.25	1.21	1.18	1.15	1.13	1.10	1.08	1.06
N=300	2.51	2.13	1.94	1.81	1.69	1.61	1.54	1.47	1.42	1.38	1.34	1.30	1.27	1.24	1.21	1.19	1.16	1.14	1.12	1.10
N=200	2.55	2.14	1.95	1.82	1.71	1.63	1.56	1.50	1.45	1.41	1.37	1.34	1.31	1.29	1.28	1.26	1.24	1.22	1.21	1.22
N=100	2.59	2.22	2.02	1.87	1.78	1.72	1.68	1.63	1.61	1.62	1.62	1.58	1.55	1.51	1.43	1.35	1.26	1.17	1.11	0.99

Table 2(b) Standard deviation

Scale	1	2	3	4	5	6	7	8	9	10	11	12	13	14	15	16	17	18	19	20
N=2000	0.03	0.03	0.03	0.03	0.03	0.04	0.04	0.04	0.04	0.05	0.05	0.05	0.05	0.05	0.05	0.06	0.06	0.06	0.06	0.06
N=1000	0.06	0.04	0.04	0.04	0.05	0.06	0.06	0.06	0.07	0.07	0.07	0.07	0.07	0.08	0.08	0.08	0.09	0.09	0.09	0.09
N=500	0.12	0.07	0.07	0.07	0.08	0.09	0.09	0.09	0.10	0.11	0.11	0.11	0.12	0.12	0.13	0.13	0.14	0.14	0.15	0.15
N=300	0.20	0.12	0.11	0.10	0.11	0.12	0.13	0.13	0.15	0.15	0.16	0.17	0.18	0.19	0.20	0.21	0.22	0.23	0.24	0.25
N=200	0.31	0.18	0.16	0.15	0.16	0.16	0.17	0.18	0.21	0.22	0.24	0.25	0.27	0.29	0.33	0.35	0.37	0.39	0.43	0.50
N=100	0.52	0.42	0.36	0.31	0.35	0.37	0.41	0.45	0.53	0.62	0.70	0.72	0.77	0.85	0.88	0.90	0.95	0.96	0.97	0.93

Table 2(c) Probability of measure failure

Scale	1	2	3	4	5	6	7	8	9	10	11	12	13	14	15	16	17	18	19	20
N=2000	0.0%	0.0%	0.0%	0.0%	0.0%	0.0%	0.0%	0.0%	0.0%	0.0%	0.0%	0.0%	0.0%	0.0%	0.0%	0.0%	0.0%	0.0%	0.0%	0.0%
N=1000	0.0%	0.0%	0.0%	0.0%	0.0%	0.0%	0.0%	0.0%	0.0%	0.0%	0.0%	0.0%	0.0%	0.0%	0.0%	0.0%	0.0%	0.0%	0.0%	0.0%
N=500	0.0%	0.0%	0.0%	0.0%	0.0%	0.0%	0.0%	0.0%	0.0%	0.0%	0.0%	0.0%	0.0%	0.0%	0.0%	0.0%	0.0%	0.0%	0.0%	0.0%
N=300	0.0%	0.0%	0.0%	0.0%	0.0%	0.0%	0.0%	0.0%	0.0%	0.0%	0.0%	0.0%	0.0%	0.0%	0.0%	0.0%	0.0%	0.0%	0.0%	0.0%
N=200	0.1%	0.0%	0.0%	0.0%	0.0%	0.0%	0.0%	0.0%	0.0%	0.0%	0.0%	0.0%	0.0%	0.0%	0.0%	0.0%	0.0%	0.0%	0.1%	0.0%
N=100	7.2%	0.8%	0.0%	0.1%	0.0%	0.2%	0.2%	0.2%	0.8%	1.8%	3.3%	6.5%	8.6%	13.4%	18.7%	25.0%	29.1%	33.5%	35.5%	39.5%

Table 3. p value from t tests for comparing entropies for Gaussian distributed 1/f noise and entropies for Gaussian distributed white noise

Scale	1	2	3	4	5	6	7	8	9	10	11	12	13	14	15	16	17	18	19	20
N=2000	0.00	0.00	0.00	0.00	0.00	0.00	0.00	0.00	0.00	0.00	0.00	0.00	0.00	0.00	0.00	0.00	0.00	0.00	0.00	0.00
N=1000	0.00	0.00	0.00	0.00	0.00	0.00	0.00	0.00	0.00	0.00	0.00	0.00	0.00	0.00	0.00	0.00	0.00	0.00	0.00	0.00
N=500	0.00	0.00	0.00	0.00	0.00	0.00	0.00	0.00	0.00	0.00	0.00	0.00	0.00	0.00	0.00	0.00	0.00	0.00	0.00	0.00
N=300	0.00	0.00	0.00	0.00	0.00	0.00	0.00	0.00	0.00	0.00	0.00	0.00	0.00	0.00	0.00	0.00	0.00	0.00	0.00	0.00
N=200	0.00	0.86	0.00	0.00	0.00	0.00	0.00	0.00	0.00	0.00	0.00	0.00	0.00	0.00	0.00	0.00	0.00	0.00	0.00	0.00
N=100	0.00	0.00	0.00	0.00	0.00	0.00	0.00	0.00	0.00	0.00	0.00	0.00	0.00	0.00	0.00	0.00	0.01	0.00	0.02	0.01
